# Incidence and Predictors of Hypoattenuated Thickening and Device‐Related Thrombus at Three Months Postprocedural CT Assessment Following Left Atrial Appendage Occlusion With Amplatzer Devices—A Single‐Center Cohort

**DOI:** 10.1002/ccd.70421

**Published:** 2025-12-09

**Authors:** Pierre Guilleminot, Carole Richard, Antoine Roger, Marlène Daller, Gabriel Laurent, Catherine Vergely, Charles Guenancia, Thibaut Pommier

**Affiliations:** ^1^ Université Bourgogne Europe CHU Dijon Bourgogne, Service de Cardiologie, PEC2 UR 7460 Dijon France; ^2^ Université Bourgogne Europe CHU Dijon Bourgogne, Service de Cardiologie Dijon France

**Keywords:** device‐related thrombus, hypoattenuation thickening, ICE, LAAO, peridevice leakage

## Abstract

**Background:**

Left atrial appendage occlusion (LAAO) has become a valuable alternative to long‐term anticoagulation for stroke prevention in patients with non‐valvular atrial fibrillation (AF), especially in those at high bleeding risk. Hypoattenuated thickening (HAT) and device‐related thrombus (DRT) remain notable postprocedural concerns. Identifying reliable predictors is essential to optimize post‐LAAO management.

**Aims:**

The aim of this study was to assess the incidence of HAT and DRT at 3 months following percutaneous LAAO and to identify clinical, anatomical, and procedural predictors—based on CT imaging evaluation—that may guide optimization of postprocedural antithrombotic strategies.

**Methods:**

We conducted a retrospective single‐center study including adult patients who underwent percutaneous LAAO with Amplatzer Amulet or ACP devices at Dijon University Hospital between April 2016 and May 2024, with available pre‐ and 3‐month postprocedural CT scans. Baseline clinical, echocardiographic, biological, procedural, and anatomical data were collected. The primary objective was to determine the incidence and predictors of HAT and DRT at 3 months.

**Results:**

Among 102 patients (mean age 76 ± 7 years, mean CHADS‐VASc 4.4 ± 1.4), HAT was observed in 25 (24%), and DRT in 3 (2.9%). Female sex and prior stroke/TIA were significantly associated with HAT occurrence. Importantly, patients discharged on dual antiplatelet therapy (DAPT) demonstrated a markedly lower incidence of HAT/DRT, suggesting a protective effect. In contrast, no anatomical parameter, including the Cressa classification, predicted thrombotic events. While Cressa morphology correlated with procedural complexity, it had no value for thrombotic risk stratification.

**Conclusions:**

This study highlights the absence of anatomical predictors of HAT/DRT, emphasizing instead clinical determinants and the protective role of DAPT. These findings support tailoring antithrombotic therapy after LAAO according to individual clinical risk profiles, particularly in patients with prior stroke/TIA or women, who may benefit from reinforced preventive strategies. Larger multicenter studies are needed to refine postprocedural management algorithms and improve risk stratification beyond anatomical assessment.

## 1 Introduction

Left atrial appendage occlusion (LAAO) has emerged as an effective alternative to long‐term oral anticoagulation for stroke prevention in patients with non‐valvular atrial fibrillation (AF), particularly those at high bleeding risk or with contraindications to anticoagulation therapy [1]. While pivotal trials such as PROTECT AF and PREVAIL established the clinical efficacy and safety of LAAO compared with warfarin [2, 3, 4], current attention increasingly focuses on device‐related findings detected during follow‐up, notably hypoattenuated thickening (HAT) and device‐related thrombus (DRT).

Both HAT and DRT, typically identified on cardiac CT or transesophageal echocardiography, have been associated with potential thromboembolic risk [5, 6]. Reported incidence rates of HAT and DRT vary across studies, reflecting heterogeneity in populations, device types, imaging protocols, and follow‐up duration [7]. Importantly, the identification of reliable predictors of HAT and DRT is critical for refining patient selection, optimizing procedural strategies, and guiding postprocedural management, including antithrombotic therapy [8].

Because the left atrial appendage (LAA) is anatomically complex and represents the primary site of thrombus formation in AF [9, 10, 11], anatomical features (such as orifice size, depth, or lobe configuration) have been proposed as potential determinants of HAT and DRT [12]. In this context, the Cressa classification, an intuitive and simplified anatomical scoring system based on standard imaging, was developed to support procedural planning and potentially anticipate adverse events [13]. However, its predictive value regarding post‐LAAO thrombotic complications remains insufficiently documented.

This single‐center retrospective study aims to assess the incidence and predictors of HAT and DRT at 3‐month follow‐up after LAAO using Amulet or ACP devices, with a specific focus on evaluating the contribution of the Cressa anatomical classification.

## 2 Materials and Methods

### 2.1 Study Design and Patients

Adult patients who underwent percutaneous LAAO at the University Hospital of Dijon (France) between April 2016 and May 2024 were screened for inclusion in this retrospective study.

Inclusion criteria were patient age ≥ 18 years, LAAO with ACP or Amulet (Abbott Cardiovascular), and available pre‐and 3 months postprocedural CT‐scan. Exclusion criteria were age < 18 years, non‐consenting patients, LAAO performed with the Watchman device (Boston Scientific), cases with missing CT data, incomplete procedural records, or unsuitable imaging quality. To address potential selection bias, baseline characteristics of excluded patients were qualitatively reviewed and appeared similar to those of included patients.

### 2.2 Data Collection

All data were extracted from electronical medical files of the University Hospital of Dijon. The following baseline patient characteristics were collected: demographics (age, sex), cardiovascular risk factors (smoking, hypertension, overweight, diabetes, dyslipidemia), comorbidities such as prior stroke, TIA, MI, heart failure. The type of AF (paroxysmal, persistent, or permanent), CHADS‐VASc, pre‐ and postprocedural anti‐thrombotic regimen, as well as echocardiographic parameters (LVEF and left atrial volume), biological parameters (GFR, hemoglobin, platelet count, NT‐proBNP) were collected.

Periprocedural and early postprocedural complications were collected during hospitalization, including pericardial effusion, tamponade, prosthesis migration, stroke, and vascular complications.

CT data were analyzed using 3mensio Structural Heart 10.6 software (Pie Medical Imaging B.V.). The anatomical classification was determined independently by two LAAO operators. If there was a discrepancy, the operators studied the scan together to determine the final classification. The same process was used to analyze scans at 3 months by two cardiac imagers. Interobserver agreement was not formally calculated, as individual readings were not recorded separately; assessments were performed visually and reconciled jointly. Therefore, kappa statistics are not available.

### 2.3 Device‐Related Complication Definitions

#### 2.3.1 HAT

HAT is defined as a hypoattenuating layer visualized on cardiac CT over the atrial‐facing surface of the LAAO device. In this study, only grade 1 of the Alkhouli classification was defined as HAT [14]. HAT is further classified as: Low‐grade HAT: a thin hypoattenuated layer with a thickness < 3 mm, without mass effect. High‐grade HAT: a more pronounced hypoattenuated layer with a thickness ≥ 3 mm. It is considered a potential indicator of delayed device endothelialization or thrombus formation. The clinical implications of HAT remain under investigation, though high‐grade forms may carry a higher thromboembolic risk [15, 16].

#### 2.3.2 DRT

DRT is defined on cardiac CT as a well‐demarcated hypoattenuated mass adherent to the surface of the LAA occlusion device, distinct from the more diffuse appearance of HAT. It is typically associated with a mass effect protruding into the left atrial cavity and may reflect organized thrombus. In this study, DRT corresponds to grades 3 and 4 of the HAT classification according to Alkhouli et al. [14]. DRT is considered a high‐risk finding due to its association with thromboembolic events [17, 18, 19].

#### 2.3.3 Peridevice Leakage (PDL)

PDL refers to residual blood flow between the occluder and the LAA wall, indicating incomplete sealing. It is typically assessed by color Doppler on transesophageal echocardiography or by contrast‐enhanced cardiac CT. PDL is categorized based on the maximum width of the leak: Minimal or small PDL < 3 mm; moderate PDL: 3–5 mm; significant PDL: > 5 mm. While small leaks (< 3 mm) are generally considered clinically acceptable, persistent or late‐onset PDL has been associated with higher thromboembolic risk and may evolve over time [20, 21, 22].

### 2.4 LAA Morphological Classification

**Figure 1:**
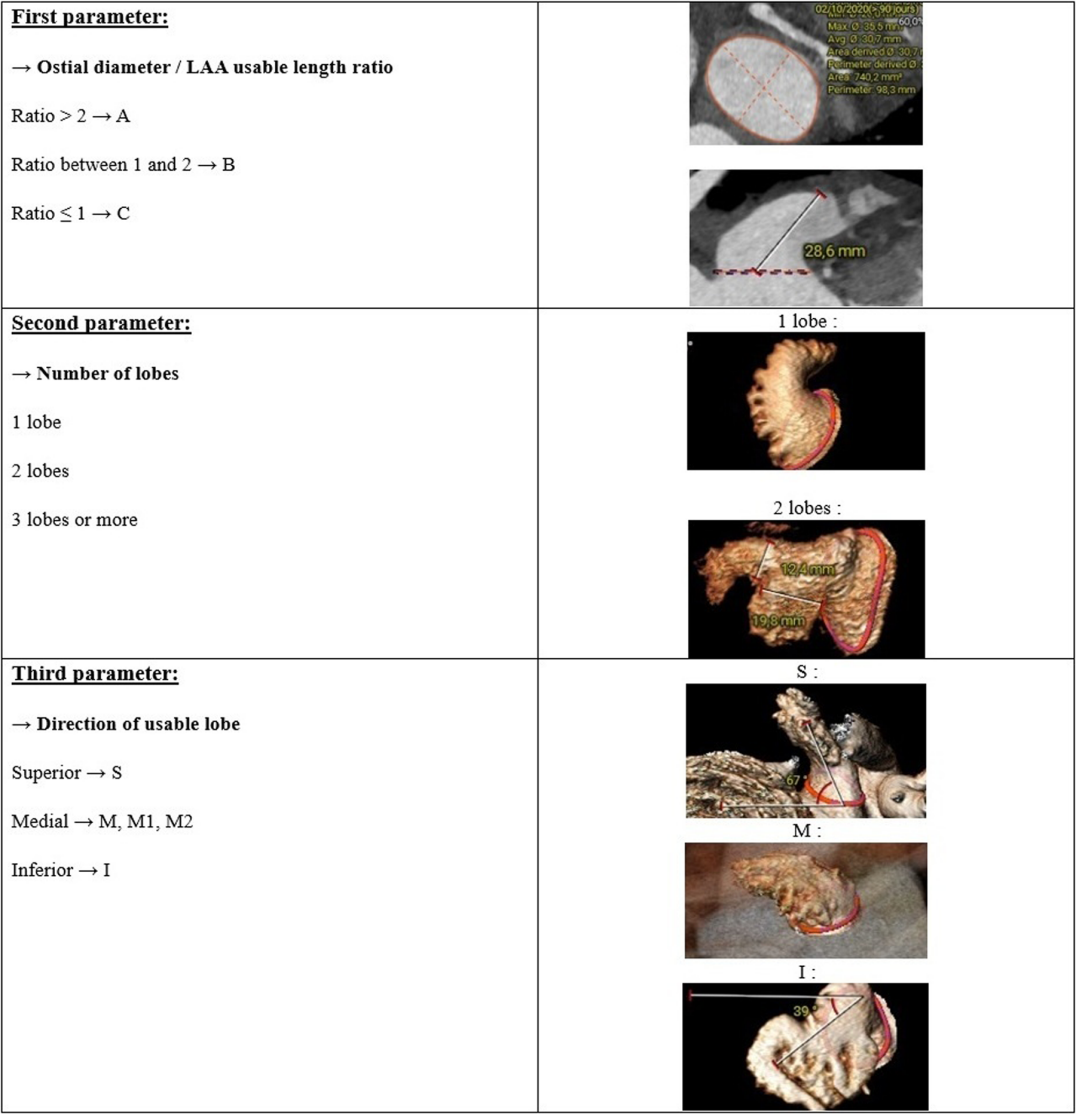
Cressa morphological classification parameters of the left atrial appendage (LAA). [Color figure can be viewed at wileyonlinelibrary.com]

The practical anatomical classification proposed by Cressa et al. involves studying three morphological parameters of the LAA, which can then be classified according to these parameters. Figure 1 illustrates representative LAA morphologies according to the Cressa classification, highlighting ostium size, number of lobes, and orientation of the main lobe to facilitate procedural planning. First, the ostial diameter relates to the effective length of the LAA. The diameter of the anatomical ostium can be measured using either the mean diameter or the diameter derived from the perimeter. If the ratio is greater than 2, the letter A is assigned. If the ratio is between 1 and 2, the letter B is assigned; if the ratio is less than or equal to 1, the letter C is assigned. Next, a number is assigned according to the number of lobes. If there is a single lobe corresponding to the body of the LAA, the number 1 is assigned. In the case of two lobes, the number 2 is assigned and 3 in case of three lobes or more. Third, a letter is assigned according to the direction of the useful lobe: S for superior, M for medial, and I for inferior. In the case of a medial lobe, “M1” is specified in the case of early 90° angulation (< 20 mm) of the useful lobe, “M2” in the case of early (< 15 mm) secondary non‐useful lobes, “M” in the case of a single lobe without 90° angulation.

### 2.5 Statistical Analysis

Continuous variables were expressed as mean ± SD or median with interquartile range (IQR) and compared using unpaired Student's *t*‐test or nonparametric Mann–Whitney *U* test, respectively. Normality of distribution of continuous variables was analyzed using the Kolmogorov−Smirnov test. Categorical variables were reported as absolute numbers and percentages and compared using the chi‐square test or Fisher's exact test. A two‐tailed *p* value of < 0.05 was considered statistically significant. A post hoc power calculation was performed to estimate the study's ability to detect associations with HAT (*n* = 25) and DRT (*n* = 3) in the sample (*n* = 102). Estimated power was 35%–40% for HAT and 15%–20% for DRT, indicating limited ability to detect small‐to‐moderate differences; this limitation is acknowledged in the Discussion. All analyses were performed using SPSS 27.0 0 software (IBM Corp., Armonk, NY, USA).

### 2.6 Ethics

According to institutional policy, approval from our Institutional Review Board was not required, and this study was completed in accordance with the Helsinki Declaration as revised in 2013.

## 3 Results

Of the 259 consecutive patients who underwent LAAO between April 2016 and May 2024, 124 had both CT scan available for analysis. Of these patients, 102 underwent the procedure with Amplatzer devices.

HAT was observed in 25 patients (24%), while DRT was identified in three patients (2.9%). Combined HAT or DRT was present in 28 patients (27%) (Table 1).

**Table 1:** Baseline characteristics according HAT and DRT status.

	HAT	No HAT		DRT	No DRT		DRT + HAT	No DRT no HAT	
*n* (%)	25 (24)	77 (76)	*p* value	3 (2.9)	99 (97.1)	*p* value	28 (27)	74 (73)	*p* value
*Patients characteristics*									
Age (years)	79 ± 4.8	77 ± 6.7	0.209	78 ± 2	77 ± 7	0.848	79 ± 7	77 ± 7	0.2
Female sex	14 (56)	24 (31.2)	0.026	1 (33.3)	37 (37.4)	0.887	15 (53.6)	23 (31.1)	0.036
Diabetes	7 (28)	22 (28.9)	0.928	0 (0)	29 (29.6)	0.555	7 (25)	22 (30.1)	0.609
Dyslipidemia	14 (60.9)	41 (54.7)	0.6	2 (66.7)	53 (55.8)	1	16 (61.5)	39 (54.2)	0.516
Current smoker	3 (12.5)	4 (5.4)	0.357	0 (0)	7 (7.4)	1	3 (11.1)	4 (5.6)	0.39
BMI (kg/m²)	28 ± 5.9	27 ± 4.8	0.527	27 ± 1	27 ± 5	0.939	27 ± 6	27 ± 5	0.523
Hypertension	23 (92)	61 (80.3)	0.174	2 (66.7)	82 (83.7)	0.438	25 (89.3)	59 (80.8)	0.309
Prior stroke/TIA	21 (84)	48 (62.3)	0.044	1 (33.3)	68 (68.7)	0.244	22 (78.6)	47 (63.5)	0.147
Prior MI	2 (8)	8 (10.4)	1	0 (0)	10 (10.1)	1	2 (7.1)	8 (10.8)	0.723
Cancer	8 (32)	15 (19.5)	0.193	1 (33.3)	22 (22.2)	0.539	9 (32.1)	14 (18.9)	0.154
Heart failure	1 (4)	9 (11.7)	0.444	0 (0)	10 (10.1)	1	1 (3.6)	9 (12.2)	0.278
PAD	6 (24)	11 (14.3)	0.257	1 (33.3)	16 (16.2)	0.425	7 (25)	10 (13.5)	0.232
CHADS‐VASc > 4	22 (88)	53 (70.7)	0.083	2 (66.7)	73 (75.3)	1	24 (85.7)	51 (70.8)	0.123
Preprocedural OAC	6 (24)	19 (24.7)	0.946	2 (66.7)	23 (23.2)	0.148	8 (28.6)	17 (23)	0.557
AF classification									
Permanent/persistent	9 (47.4)	27 (47.4)	1	2 (100)	34 (45.9)	0.221	11 (52.4)	25 (45.5)	0.589
Procedural features									
US guiding (ICE/TEE)	17 (68)	54 (70.1)	0.539	2 (66.7)	69 (69.7)	0.935	19 (67.9)	52 (70.3)	0.482
PDL	5 (20)	19 (24.7)	0.632	1 (33.3)	23 (23.2)	0.557	6 (21.4)	18 (24.3)	0.758
*Biological parameters*									
Hemoglobin (g/dL)	13.3 ± 1.8	13.2 ± 1.7	0.746	14.7 ± 0.3	13.2 ± 1.7	0.135	13.5 ± 1.7	13.1 ± 1.7	0.381
Platelet count (G/L)	221 ± 76	226 ± 73	0.816	207 ± 45	225 ± 74	0.676	220 ± 73	226 ± 74	0.701
GFR (mL/min/m²)	61 ± 24	67 ± 38	0.412	72 ± 29	66 ± 35	0.743	62 ± 24	67 ± 38	0.508
NT proBNP (µmol/L)	1123 ± 1775	1448 ± 1627	0.496	858 ± 267	1387 ± 1678	0.659	1093 ± 1671	1472 ± 1655	0.409
Antithrombotic at discharge									
DAPT	9 (39.1)	43 (57.3)	0.126	0 (0)	52 (54.7)	0.061	9 (34.6)	43 (59.7)	0.028
TTE parameters									
LVEF (%)	61 ± 8	61 ± 6	0.964	70 ± 7	61 ± 7	0.055	62 ± 8	61 ± 6	0.472
LA volume (mL/m²)	52 ± 25	50 ± 18	0.812	75 ± 0	50 ± 20	0.236	54 ± 25	49 ± 17	0.492

*Note:* Data are presented as *n* (%) or mean ± SD.

Abbreviations: BMI, body mass index; DAPT, dual antiplatelet therapy; GFR, glomerular filtration rate; ICE, intracardiac echocardiography; LA, left atrial; LVEF, left ventricular ejection fraction; MI, myocardial infarction; PAD, peripheral artery disease; TEE, transesophageal echocardiography; TTE, transthoracic echocardiography; US, ultrasound.

Patients who developed HAT were more frequently female (56% vs. 31%, *p* = 0.026) and had a higher prevalence of prior stroke or TIA (84% vs. 62%, *p* = 0.044). There was also a trend toward a higher occurrence of HAT in patients with a CHADS‐VASc score > 4 (88% vs. 70.7%, *p* = 0.083), although this difference did not reach statistical significance. Patients discharged on dual antiplatelet therapy (DAPT) were less likely to develop combined HAT or DRT (34.6% vs. 59.7%, *p* = 0.028). Other baseline characteristics, including age, diabetes, hypertension, dyslipidemia, and renal function, were similar between groups and are therefore not detailed here.

Regarding anatomical predictors, no LAA were classified as type A. Types B and C exhibited HAT rates of 29.1% and 19.1%, respectively (*p* = 0.260), and DRT rates of 0% and 6.4% (*p *= 0.094), without statistical significance. The absence of type A morphologies may reflect the small sample size or limitations in the Cressa classification. No significant associations were observed between the first, second, or third Cressa parameters and the occurrence of HAT or DRT (Table 2).

**Table 2:** Impact of morphological classification on early CT‐scan evaluation and procedural data.

	Cressa morphological classification												Total
	1st parameter (ostium size)				2nd parameter (number of lobes)				3rd parameter (orientation of the main lobe)				
	A	B	C	*p* value	1	2	3	*p* value	M	S	I	*p* value	
*n* (%)	0 (0)	55 (53.9)	47 (46.1)		68 (66.7)	29 (28.4)	5 (4.9)		65 (63.7)	26 (25.5)	11 (10.8)		102
*Devices analysis*													
HAT	0 (0)	16 (29.1)	9 (19.1)	0.260	15 (22.1)	9 (31)	1 (20)	0.624	15 (23.1)	7 (26.9)	3 (27.3)	0.905	25
PDL	0 (0)	13 (23.6)	11 (23.4)	0.978	18 (26.5)	5 (17.2)	1 (20)	0.607	14 (21.5)	2 (18.2)	8 (30.8)	0.584	24
DRT	0 (0)	0 (0)	3 (6.4)	0.094	3 (4.4)	0 (0)	0 (0)	0.462	3 (4.6)	0 (0)	0 (0)	0.415	3
Procedural features													
Procedural success	0 (0)	55 (100)	47 (100)	—	68 (100)	29 (100)	5 (100)	—	26 (100)	65 (100)	11 (100)	—	102
Procedural time	—	86 ± 26	94 ± 28	0.137	86 ± 22	97 ± 31	93 ± 44	0.225	86 ± 28	88 ± 19	109 ± 27	0.045	—
Fluoroscopy time	—	17 ± 10	18 ± 12	0.563	16 ± 10	21 ± 12	17 ± 14	0.101	17 ± 11	15 ± 7	24 ± 11	0.048	—
Total procedural complication	0 (0)	4 (7.3)	4 (8.5)	0.551	5 (7.4)	3 (10.3)	0	0.705	6 (9.2)	2 (18.2)	0 (0)	0.134	8

*Note:* Data are presented as *n* (%) or mean ± SD.

Abbreviations: DRT, device‐related thrombus; HAT, hypoattenuated thickening; PDL, peridevice leak.

Cressa classification parameters: 1st parameter—ostium size (A: ratio > 2, B: 1–2, C: ≤ 1); 2nd parameter—number of lobes (1, 2, or ≥ 3); 3rd parameter—orientation of the main lobe (S: superior, M: medial, I: inferior, with subcategories M1/M2 for early angulation or secondary lobes).

All anatomical subtypes showed 100% procedural success. Procedural and fluoroscopy times tended to be longer in type C morphologies, particularly with the third Cressa parameter (procedural time: 109 ± 27 min, *p* = 0.045; fluoroscopy time: 24 ± 11 min, *p* = 0.048), but these differences did not translate into a higher rate of procedural complications.

Overall, the incidence of HAT and DRT remained relatively low. Clinical characteristics, namely female sex, prior stroke/TIA, and DAPT, were the main predictors of HAT, while morphological classification did not significantly predict thrombotic complications.

## 4 Discussion

### 4.1 Main Findings

In this retrospective single‐center study, we report a 24% incidence of HAT and a 2.9% incidence of DRT at 3 months following LAAO with Amulet or ACP devices. These results align with prior reports: Cochet et al. [23] and Iriart et al. [16] described HAT prevalence ranging from 15% to 40%, while DRT rates remain consistently low across studies, typically between 3% and 4% [5, 24]. Interestingly, we identified female sex and prior stroke or TIA as significant predictors of HAT, which may reflect sex‐related differences in endothelialization, atrial substrate, or thrombogenicity, and the impact of prior cerebrovascular events on platelet and coagulation profiles [25, 26].

Notably, patients discharged on DAPT showed a lower incidence of combined HAT or DRT, supporting prior observations that optimized postprocedural antithrombotic therapy reduces thrombotic risk [5, 27]. Compared with single antiplatelet or anticoagulant strategies, DAPT may enhance endothelial coverage and prevent thrombus formation in high‐risk patients [28].

However, our study did not demonstrate a significant association between the Cressa anatomical classification and device‐related complications. While types B and C showed numerically different rates of HAT and DRT, these differences were not statistically significant, consistent with Kramer et al. [29]. The limited predictive value may relate to the fact that the Cressa classification primarily guides procedural planning rather than directly capturing the biological processes leading to HAT/DRT. The Cressa classification is based solely on the shape and orientation of the LAA. However, the risk of HAT or DRT also depends on clinical factors (such as sex, prior stroke/TIA, and antithrombotic therapy) and biological factors (including coagulation status and endothelial function). Therefore, to better predict these complications, it may be useful to combine anatomical data with clinical and biological characteristics, or to use alternative imaging modalities like intracardiac echocardiography or 4D CT that provide more detailed information on blood flow and endothelial coverage [30]. Importantly, longer procedural and fluoroscopy times in anatomies classified under the third Cressa parameter (particularly inferior‐oriented lobes) suggest the classification remains useful for anticipating procedural complexity.

### 4.2 Study Limitations

Our study has several important limitations. First, it is a retrospective, single‐center analysis with a modest sample size, which limits the generalizability of our findings. The relatively small number of events, particularly for DRT (only three cases), reduces the statistical power to detect meaningful predictors and may underestimate associations seen in larger cohorts. Unmeasured confounders, such as operator experience, device sizing, and procedural nuances, may also influence outcomes. Additionally, our analysis focused on 3‐month follow‐up imaging, and no long‐term clinical outcome data, limiting our ability to assess the true impact of HAT or DRT on stroke or embolic events over time. Finally, while CT imaging offers excellent sensitivity for detecting device‐related abnormalities, interobserver variability and evolving classification criteria [29] remain challenges that could impact reproducibility.

### 4.3 Perspectives

Despite these limitations, our study provides valuable insights. Clinically, it highlights the need to focus on individual patient risk profiles, particularly prior cerebrovascular events and sex, rather than solely anatomical features when assessing thrombotic risk after LAAO. The Cressa classification retains its practical role in procedural planning, helping operators anticipate challenges, especially for anatomies with inferiorly directed lobes where longer procedural and fluoroscopy times were observed. Future research should aim to integrate anatomical models with patient‐specific clinical and biological data to develop more robust risk prediction tools. Moreover, larger multicenter studies with longer‐term follow‐up are warranted to better define the clinical relevance of imaging‐detected HAT and to establish evidence‐based postprocedural antithrombotic strategies.

## 5 Conclusion

This study confirms that while HAT and DRT remain relatively infrequent after LAAO, their occurrence is more strongly influenced by clinical rather than anatomical factors. The Cressa anatomical classification, although not predictive of device‐related complications, continues to hold value in procedural planning. Future studies should address specific questions such as the optimal DAPT and the potential role of advanced imaging modalities for improved risk stratification. Clinically, enhanced monitoring may be warranted for female patients or those with a history of stroke/TIA. Overall, future strategies should combine anatomical, clinical, and procedural insights and prioritize long‐term follow‐up to fully understand the implications of postprocedural imaging findings.

## Conflicts of Interest

The authors declare no conflicts of interest.

## Data Availability

The data sets used and/or analyzed during the current study are available from the corresponding author on reasonable request.
